# Resource Optimization Scheme for Multimedia-Enabled Wireless Mesh Networks

**DOI:** 10.3390/s140814500

**Published:** 2014-08-08

**Authors:** Amjad Ali, Muhammad Ejaz Ahmed, Md. Jalil Piran, Doug Young Suh

**Affiliations:** Department of Electronics and Radio Engineering, Kyung Hee University, Yongin 446-701, Korea; E-Mails: ejaz629@gmail.com (M.E.A.); piran@khu.ac.kr (M.J.P.); suh@khu.ac.kr (D.Y.S.)

**Keywords:** ILP, wireless mesh networks, random topology, QoS provisioning routing, heuristic algorithm

## Abstract

Wireless mesh networking is a promising technology that can support numerous multimedia applications. Multimedia applications have stringent quality of service (QoS) requirements, *i.e.*, bandwidth, delay, jitter, and packet loss ratio. Enabling such QoS-demanding applications over wireless mesh networks (WMNs) require QoS provisioning routing protocols that lead to the network resource underutilization problem. Moreover, random topology deployment leads to have some unused network resources. Therefore, resource optimization is one of the most critical design issues in multi-hop, multi-radio WMNs enabled with multimedia applications. Resource optimization has been studied extensively in the literature for wireless *Ad Hoc* and sensor networks, but existing studies have not considered resource underutilization issues caused by QoS provisioning routing and random topology deployment. Finding a QoS-provisioned path in wireless mesh networks is an NP complete problem. In this paper, we propose a novel Integer Linear Programming (ILP) optimization model to reconstruct the optimal connected mesh backbone topology with a minimum number of links and relay nodes which satisfies the given end-to-end QoS demands for multimedia traffic and identification of extra resources, while maintaining redundancy. We further propose a polynomial time heuristic algorithm called Link and Node Removal Considering Residual Capacity and Traffic Demands (LNR-RCTD). Simulation studies prove that our heuristic algorithm provides near-optimal results and saves about 20% of resources from being wasted by QoS provisioning routing and random topology deployment.

## 1. Introduction

Wireless mesh networks (WMNs) are dynamically self-organized, self-configured, self-healing, and easy-to-install multi-hop networks. This type of networks are becoming a promising wireless technology that has great potential to provide multimedia services over large coverage areas in the future [1]. WMNs can provide the same level of network reliability, capacity, and security as wired networks. In recent years, there has been an increasing demand for quality-of-service (QoS)-demanding real-time and multimedia applications, such as audio and video, in large application areas. These QoS-demanding applications are promoted for two reasons: one is the pervasive use of computing devices, such as laptop computers, personal digital assistants, video surveillance cameras, automotive computing devices, smart phones, and wearable computers; and the other is the fast-growing deployment of multi-hop wireless mesh networks to connect these computing devices [2]. In the past, WMNs were only considered to provide “last mile” internet access, but now high capacity WMNs are being rapidly deployed in large business organizations, industries, airports, military bases, ports, and borders for remote monitoring, to locate and track high-value equipment for theft protection to provide real-time video feeds to first responders for staff safety in emergency situations, and to provide variety of other multimedia applications and services. Such WMNs are deployed and owned by individual organizations for their exploitation and their access is not publically available. We call such WMNs private WMNs (PWMNs), and it is expected that the video surveillance market for PWMNs will be a $37 billion market in 2015 [3].

In multi-hop, multi-channel WMNs, the nodes are equipped with inexpensive and readily available 802.11 multiple radios, by which they can exploit the IEEE 802.11 defined orthogonal channels to enhance the overall network capacity. However, the availability of orthogonal channels is limited, e.g., three orthogonal channels are defined for 802.11 b, and 12 orthogonal channels are defined for 802.11 a. Furthermore, interference with the adjacent nodes adversely affects the capacity of WMNs [4]. Channel assignment (CA) is a key issue in guaranteeing network connectivity and leveraging the capacity of WMNs. A WMN node must share a channel with neighboring nodes with which it wants to establish connectivity. Most of the CA schemes have been proposed to address connectivity and capacity issues of WMNs, which are mainly categories into three categories, static, dynamic, and hybrid. In [4,5] static and interference-aware CA approaches are addressed, dynamic interference-aware CA approaches are discussed in [6,7] while hybrid CA approach is presented in [8]. The hybrid and dynamic approaches are more attractive because they allow channel switching among available channels according to the packet destination, but radio switching from one channel to another channel induces further delay, which is in the range of a few hundred microseconds to a few milliseconds. Furthermore, the current IEEE 802.11 cannot guarantee a mechanism for nodes to switch radios on a packet basis. Therefore, it is reasonable to allocate the channels to the radios on the mesh backbone nodes statically or permanently [4].

Routing protocols can be categorized into two major classes in the context of the types of applications and services they support: (1) QoS provisioning routing that enables multimedia and other QoS demanding applications; (2) conventional routing that enables non-real time and multimedia applications. These two different types of routing can cause two different network problems in their respective fields of application. Conventional routing induces resource overutilization problems [9–11]. In resource overutilization, many traffic flows share the same network resources (e.g., router buffer and data channel), which may cause network congestion that reduces the overall network throughput. Designing QoS provisioning routing protocols that enable multimedia and QoS-demanding applications and services is an emerging field of research. Currently available QoS provisioning routing protocols follow a self-serving approach to find and select an end-to-end routing path that fulfills the QoS requirements of multimedia applications [12].Therefore, in QoS provisioning routing bandwidths, delays, and Packet Loss Ratio (PLR) are not only the multimedia application requirements, but they are also being used as default routing parameters [13]. Moreover, link quality metrics e.g., Expected Transmission Count (ETX), per-hop Round Trip Time (RTT), and per-hop Packet Pair delay (PktPair) are a few better known greedy parameters that also used in QoS provisioning routing. QoS provisioning routing protocols that are based on stated parameters adopt greedy selection mechanisms and create a network resource underutilization problem. In a resource underutilization problem, abundant multimedia traffic flows follows distinct routing paths and network resources (e.g., router buffer, data channel) over those routing paths may not be shared by other multimedia traffic flows. Thus, the allocated bandwidth will not be effectively utilized, which causes a resource wastage problem and results in high network deployment costs. Network congestion in wireless and mesh networks has been studied extensively in the literature [14–17], however to the best of our knowledge QoS provisioning routing-based resource underutilization has not been addressed.

The ideal deployment of WMNs requires conducting detailed site surveys to find the appropriate locations before the actual placement of the Wireless Mesh Routers (WMRs) and communication links. This practice is usually followed in academic test beds, but the practical deployment of WMNs is usually unplanned or random [18,19]. This makes them easy to deploy and reduces the network administration time, but may lead to the installation of unused physical layer resources (e.g., WMRs, communication links) that cause high deployment and operating costs. For example, if a WMR never routes through one of its neighbors, then that neighbor's link is questionable [20]. For example, if most WMRs are routed through only one or two neighbors, then it might be worth keeping only those neighbors. Although redundancy is one of the core design factors in WMNs, redundancy beyond a specific threshold wastes resources and increases the deployment and operating costs. Moreover, with the increasing bandwidth demands and the scarcity of available frequency spectrum, designing resource-efficient wireless mesh networks is a new challenge [21]. Thus, supporting multimedia applications and services by using minimum physical layer resources with overall low ownership cost is a challenge task.

To the best of our knowledge, the resource wastage problem caused by QoS provisioned routing and random deployment of WMNs have not been studied in the literature, hence, it is addressed for the first time in this paper. Our objective is to reconstruct the optimal connected mesh backbone topology with the minimum number of communication links and WMRs that satisfy the given end-to-end QoS demands for multimedia traffic and the identification of extra resources while maintaining redundancy. Designing QoS provisioning routing protocols to support emerging multimedia applications in WMNs is an open issue, and finding a feasible path is an NP-complete problem [22]. To achieve our objective, we first propose an Integer Linear Programming (ILP) based solution to obtain the optimal results; then, we propose a polynomial time heuristic algorithm called Link and Node Removal Considering Residual Capacity and Traffic Demands (LNR-RCTD).

The rest of the paper is organized as follows. In Section 2, we discuss an extensive literature survey. Section 3, outlines models, assumptions, and problem formation. The proposed ILP-based solution is presented in Section 4. Our heuristic LNR-RCTD algorithm with its complete working details is given in Section 5. Results and a performance comparison are shown in Section 6, and finally, Section 7 concludes the paper and presents possible future work.

## 2. Literature Review

Resource optimization in terms of topology control has been studied extensively in the literature. Most of the available literature on topology control has not considered the issues related to QoS-enabled WMNs. The comprehensive survey on topology control presented in [23] provides a good background for interested readers. The most fundamental issue considered in topology control is wireless network connectivity. Other than this fundamental issue, many other objectives listed below have also been addressed in the prior literature:

*Minimum Energy Consumption:* Energy consumption is a hot research topic in small battery networks such as wireless sensor and mobile *Ad Hoc* networks. In such networks, the main issue is to design a network with minimum power utilization to maximize the network's lifetime. Efficient battery management [24–26], system power management [27,28], transmission power management [29], and energy-efficient routing [30–32] are the major ways to save the energy in wireless *Ad Hoc* networks and increase their lifetime. Similarly, extensive work has been done on energy saving for wireless sensor networks by energy-efficient routing [33,34], energy-aware transmissions [35], and battery management [36,37].

*Minimum Interference:* Interference on the receiving nodes occurs when 1 hop neighboring nodes transmit their own signals over the same channel at the same time that the receiving nodes receive their signals. Interference causes collisions, which may leads to packet loss. Thus, more energy is consumed in the retransmissions of lost packets and the overall throughput degrades. The interference-aware channel assignment problem in multi-hop wireless mesh networks has been broadly discussed in the literature [37–39] to find ways of minimizing the interference.

*Quality-of-Service:* QoS-based topology control schemes aim to provide a guaranteed bandwidth has been widely considered for wireless *Ad Hoc* networks [40,41]. The primary objective of such schemes is to construct a topology that fulfills bandwidth requirements under the constraint of efficient-energy utilization. Some literature [42,43] is also available on QoS-based topology control for WMNs. The objective in WMNs is similar to construct paths those fulfill the bandwidth or delay demands of the traffic and minimize interference.

*Cost Effective Deployment:* Designing a cost-effective infrastructure-less wireless Ad Hoc and sensor networks is highly challenging due to the lack of central administration, mobility, and limited resources. Ongoing efforts are aimed at developing cost-effective algorithms for Ad Hoc and sensor networks [44]. Still, battery life is a major factor to consider when measuring the cost-effective deployment of wireless *Ad Hoc* and sensor networks [45].

Resource Optimization-Based Deployment: The minimum resource-based deployment of wireless multi-hop networks to ensures that multi-hop network topology is constructed with the help of minimum physical and MAC layer resources with minimum overall ownership cost and that no physical and MAC layer resources are being wasted. Under such directions, before the mesh node is actually placed exhaustive site surveys methodology has been proposed to construct a tightly bounded topology with minimum deployment costs [46]. Moreover, the mesh node height is calculated before its actual placement to save the physical resources and deployment costs [47].

Zhang. *et al*'s. proposed topology control for service-oriented WMNs [48] is the only work proposing service-oriented applications that is closely related to our work. The aim of their study is to build an WMN topology that only fulfill the requirements of service-oriented applications while the aim of our study is to reconstruct the optimal connected mesh backbone topology with the minimum number of physical layer communication links and WMRs that satisfy the given end-to-end QoS demands for multimedia traffic and the identification of extra physical layer resources while maintaining redundancy.

## 3. System Model and Problem Formation

In this section, we first describe models, assumptions, and calculations used throughout the paper; and then we formulate our problem. We divided notations into two tables; Tables 1 and 2.

Table 1 presents the notations used in System Model, Interference Model, Assumptions, Calculations, Problem Formation, and ILP-Based Solution while Table 2 presents the notations used in LNR-RCTD Algorithm.

### 3.1. System Model

**Figure 1.**
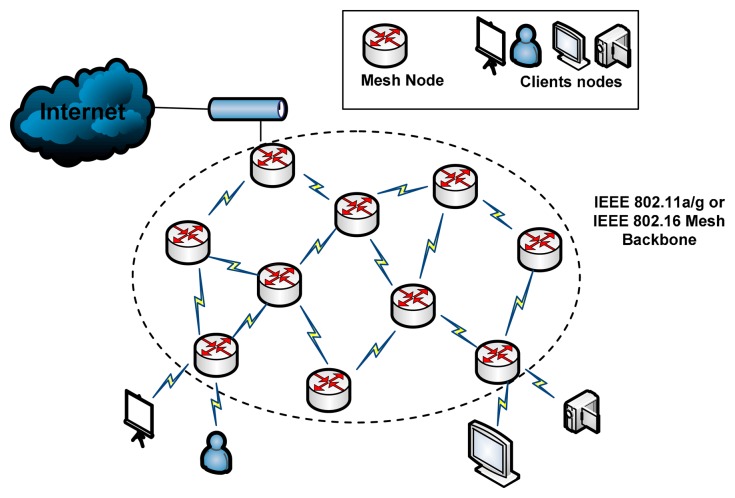
Wireless mesh backbone network architecture.

Multi-hop WMNs (MWMNs) consist of a number of stationary WMRs, forming a wireless backbone. Some WMRs serve as access points (APs) for wireless multimedia users while remaining are serving as wireless mesh backbone relay routers only; as shown in Figure 1. Each WMR *v_i_* is equipped with certain number of hybrid IEEE 802 radios denoted as R_vi_. One radio can work on one channel during the assignment process. The set of orthogonal channels available in the network is denoted as *C*, where *C* = {1,2,… . ,*k_max_*}. The network topology can be modeled as an undirected graph *G* = (*V*, *E*), where *V* is the set of mesh nodes and *E* is the set of links. There is link *e*_(_*_i_*_,_*_j_*_)_ between WMR *v_i_* and *v_j_* if their Euclidean distance is smaller than the transmission range, assuming that all the WMRs have the same transmission ranges. Thus, multi-channel and multi-radio wireless backbone mesh topology provides multiple paths for each source and destination multimedia users.

### 3.2. Interference Model

We use the interference range model [49], which is a special case of the protocol model [50]. Similar model with conjunction to Request to Send/Clear to Send (RTS/CTS) has been used in [51] to form a WMN. Interference range model states that link *e_i,j_* between nodes *v_i_* and *v_j_*, and link *e_k,l_* between nodes *v_k_* and *v_l_* could not be assign same channel if the sender or receiver of one of them is in the interference range of the sender or receiver of the other one; more specifically, if *d_vi_*.*_vk_*, *d_vi_*,*_vl_*, *d_vj,vk_*, *d_vj,vl_* ≤ *I_R_*, where *d_vi_*.*_vk_*, *d_vi_*,*_vl_*, *d_vj,vk_*, and *d_vj,vl_* is Euclidean distance between subscripted nodes. Two links out of their interference ranges can be assigned the same channel.

### 3.3. Assumptions and Calculations

#### 3.3.1. Traffic Characteristics

We consider only unicast traffic that contains a single source and single destination. QoS provisioned paths are computed for each single flow using APAC [52]. Thus, each end-to-end path is attached with each unicast flow. The end-to-end path for each flow remains constant.

#### 3.3.2. Close Bounded Network

The maximum numbers of traffic flows are known, similar to [43]. The traffic demand is static, as private WMNs are assumed to be used only by their permanent or regular clients and not shared or used by other public users.

#### 3.3.3. Residual Link Capacity

Channels are time-slotted and multiple users can share a single channel. Thus, the residual capacity of any link/channel can be calculated by subtracting the total aggregated used capacity from its total assigned capacity given as below:

(1) $$RC_{e_{i,j}}=CC_{e_{i,j}}-\sum_{f_{o}=1}^{F_{e_{i,j}}^{\prime }}f_{o}$$

#### 3.3.4. Delay and Packet Loss Calculations

Each WMR computes its delay and PLR periodically with its all one-hop neighbors by sending the Hello packets, according to Equations (2) and (3), respectively. Every node stores these computed values locally and forward these values to source node during path finding process. Finally, source node calculates the end-to-end delay and PLR of each path according to Equations (4) and (5), respectively:

(2) $$D_{WMR_{j}}=D_{q,j}(t)+D_{l,j}+D_{p,j}$$

where *D_WMRj_* is the total estimated delay at node *j* at any particular time *t*, which is based on queuing delay *D_q,j_*(*t*), link delay *D_l,j_*, and propagation delay *D_p,j_*:

(3) $$PLR_{WMR_{j}}=\frac{L_{q,j}(t)\times L_{CH,j}(t)}{L_{q,j}(t)\times L_{CH,j}(t)+NPRS}$$

where *PLR_WMRj_* is the total estimated PLR at node *j* at any particular time *t*, which is based on packet loss value due to buffer overflow *L_q,j_*(*t*) and packet loss value due to channel conditions *L_CH,j_*(*t*).*NPRS* is the number of packet received successfully:

(4) $$\tau {\left(t\right)}_{p}=\sum_{j=0}^{n}D_{WMR_{j}}$$

where *τ*(*t*)*_p_* is the total estimated end-to-end delay of path *p* at any particular time *t* and *n* is the total number of network nodes in path *p*:

(5) $$l{\left(t\right)}_{p}=1-\prod_{j=0}^{n}(1-PLR_{WMR_{j}})$$

where *l*(*t*)*_p_* is the total estimated end-to-end *PLR* of path *p* at any particular time *t* and *n* is the total number of network nodes in path *p*.

### 3.4. Problem Statement and Formation

The problem is to reconstruct the optimal mesh backbone topology and identify extra physical layer resources for multimedia-enabled WMN modeled as an undirected graph *G* = (*V*, *E*) where, *V* = {*v*_1_, *v*_2_, *v*_3_, … ., *v_n_*} is the set of the WMRs and *E* = {*e*_1_, *e*_2_, *e*_3_, … . , *e_m_*} is the set of the edges among WMRs, *e_i_* is a link between two WMRs say *v_i_* and *v_j_* whose Euclidean distance is less than their transmission ranges; thus, the link *e_i_* could also be represented as *e*_(_*_i,j_*_)_. *I*_(_*_vi_*_)_ is the set of incident edges of any WMR *v_i_* where, *F* = {*f*_1_, *f*_2_, *f*_3_, … . , *f_y_*} is the set of network flows and each flow represents a bandwidth demand. *C* = {*c*_1_, *c*_2_, *c*_3_, … . , *c_k_*} is the set of orthogonal channels. A central static/ permanent channel allocation approach to minimize the interference and maximize the network capacity similar to [4] is adopted. QoS-based routing is considered separately to channel assignment. Static channel allocation is given as matrix *a*_(_*_i,j_*_),_*_k_* of *m*_(_*_i,j_*_)_ * *k* size, where *m* is the total number of edges and *k* is the total number of channels, where matrix value can be either 0 or 1 showing whether a channel is assigned to an edge or not. A comprehensive survey on channel assignments techniques was done in [53], which provides good references and background for interested readers. *CC* = {*cc_e_*_1_, *cc_e_*_2_, c*c_e_*_3_, … . , *cc_em_*} is the set of channel capacities where *cc_e_*_(_*_i,j_*_)_ is the total capacity assigned to any link *e_i,j_* and *R_c_* = {*r_e_*_1_, *r_e_*_2_, *r_e_*_3_ … . *r_em_*} is the set of residual capacities of each link. All possible end-to-end paths for all flows are known and *p_fy_* = {*p*_1_, *p*_2_, *p*_3_ … . *p_z_*} is the set of all possible paths for any flow *f_y_, D* = {*d_f_*_1_, *d_f_*_2_, *d_f_*_3_, …*d_fy_*} is the set of maximum affordable delay and *PLR* = {*P_lf_*_1_, *Pl_f_*_2_, *Pl_f_*_3_, … *Pl_fy_*} is the set of *PLR* requirement of network flows *f_y_*.

## 4. ILP-Based Solution

In this section, we present our ILP-based optimization solution. The problem is to reconstruct an optimal connected mesh backbone topology for multimedia traffic with minimum number of communication links and WMRs. Therefore, the objective in Equation (7) is to minimize the communication links by eliminating the underutilized links by routing their respective multimedia traffic flows on new routes to improve the utilization of active communication links. Finally, a WMR is identified as extra if sum of their incident links become zero.

### 4.1. Decision Variable

(6) $$T(e_{i,j},c_{k})=\{\begin{array}{c} 1\\ 0 \end{array}\}$$

The value is 1 if link *e_i,j_* remains in the networks using channel *c_h_* or 0 otherwise.

### 4.2. Objective Function

Our objective function is to minimize:

(7) $$\text{Minimize Z}=\sum_{f_{o}\in F}\sum_{e_{i,j}\in E}\sum_{c_{h}\in C}T(e_{i,j},c_{h})$$

Subject to the following constraints:

### 4.3. Link Capacity Constraint

The sum of all the flows on any link *e_i,j_* must be less than or equal to the total assigned capacity to that link:

(8) $$\begin{array}{c} \sum_{f_{o}\in F}T(e_{i,j},c_{h})*f_{o}*a_{(i,j),k}\leq CC_{e_{i,j}}\\ \forall e_{i,j}\in E\;\text{and}\;\forall c_{h}\in C \end{array}$$

### 4.4. Flow Conservation Constraint

The sum of all flows on any link *e_i,j_* that is to be removed must be less than or equal to the sum of residual capacities over all incident links:

(9) $$\sum_{f_{o}\in F}T(e_{i,j},c_{h})*f_{o}*a_{(i,j),k}-\sum_{e_{i,j}\in I_{\left(i\right)}}T(e_{i,j},c_{h})*r_{e_{i,j}}\leq 0$$

### 4.5. Routing Constraint

A flow cannot pass through a link that is not in the set of its valid paths and each flow can traverse through only a single path:

(10) $$\sum_{e_{i,j}\notin P_{f_{0}}}T(e_{i,j},c_{h})*f_{o}*a_{(i,j),k}=0$$

(11) $$T(e_{i,j},c_{h})*f_{o}*a_{(i,j),k}=\overset{\forall v_{j}\in V}{\underset{\forall f_{0}\in F}{T(e_{j,l},c_{h})*f_{o}*a_{(j,l),k}}}$$

### 4.6. Network Constraint

All edges that do not belong to a set of paths should be removed, and similarly, all nodes whose sum of incident links is zero should also be removed:

(12) $$\sum_{e_{i,j}\in P^{\prime }}T(e_{i,j},c_{h})*f_{0}*a_{(i,j),k}=0$$

(13) $$\underset{\forall v_{j}\in V}{\sum_{e_{i,j}\in I_{v_{j}}}T(e_{i,j},c_{h})*f_{0}*a_{(i,j),k}=0}$$

where, p′ is the set of links not used to transmit any flow and *I_vj_* is the set of incident links of any node *v_j_*.

### 4.7. Source-Destination and Influx-Outflux Constraint

The amount of any traffic flow *f*_0_ generated by a source node must be equal to the traffic received by a destination node and the amount of incoming traffic on any intermediate node *v_j_* must be equal to the outgoing flow:

(14) $$T(e_{S_{i,j}},c_{h})*f_{0}*a_{(i,j),k}=T(e_{l,D_{i}},c_{h})*f_{0}*a_{(i,j),k}$$

(15) $$\underset{\forall f_{o}\in F}{T(e_{i,j},c_{h})*f_{o}*a_{(i,j),k}=T(e_{j,l},c_{h})*f_{o}*a_{(j,l),k}}$$

### 4.8. QoS Constraint

Any flow *f_o_* encountering underutilized links in its current used path will be shifted on any one of the newly computed paths who meets its QoS requirements of end-to- end delay and PLR. The newly selected path must be underutilized link free:

(16) $$\sum_{p\in P_{f_{0}}}T(e_{i,j},c_{h})*f_{o}*\tau {\left(t\right)}_{p}*a_{(i,j),k}\leq d_{f_{0}}$$

(17) $$\underset{\forall f_{o}\in F}{\sum_{p\in P_{f_{o}}}T(e_{i,j},c_{h})*f_{o}*l{\left(t\right)}_{p}*a_{(i,j),k}\leq Pl_{f_{0}}}$$

where *d_fo_* and *Pl_fo_* are maximum affordable delay and PLR limits, respectively, of flow *f_o_*.

### 4.9. Topology Construction Constraint

The links between any two nodes are bidirectional:

(18) $$\underset{\forall e_{i,j}\in E}{e_{i,j}=e_{j,i}}$$

### 4.10. Non Negativity Constraints

All decision variables must be greater than or equal to zero:

(19) $$T(e_{i,j},c_{h})\geq 0$$

## 5. Link and Node Removal Considering Residual Capacity and Traffic Demands (LNR-RCTD)

### 5.1. LNR-RCTD Algorithm

In this section, we discuss the development of our proposed heuristic algorithm called LNR-RCTD for un-optimized and random topology WMNs. The final outcome of our proposed heuristic LNR-RCTD is the connected graph *G*′ (*V*′, *E*′) ⊆ *G* (*V*, *E*) , where *V*′ ⊆ *V* and *E*′ ⊆ *E*. Node connectivity is based on radius of the node, which is further based on the transmission power (*T_r_*) of that node. Increasing the value of *T_r_* increases the degree of node connectivity and lowering the value of *T_r_* decreases the degree of node connectivity. Therefore, *T_r_* is a design parameter that has a significant impact on constructing an energy-efficient mesh topology. In this paper, our primary objective is to reconstruct an optimal connected mesh backbone topology. Therefore, the value of *T_r_* is selected such that each WMR must be connected to atleast one other WMR.

LNR-RCTD is significantly based on the APAC algorithm for computing the available paths between any two WMRs. APAC is an efficient algorithm that keeps track of all available paths and cycles between two network nodes, but not the all visited vertices, which significantly reduces its computational complexity. We modify APAC to compute the paths based on their QoS requirements between two WMRs.

The LNR-RCTD algorithm finds underutilized network resources in multimedia and real-time enabled WMNs. It processes all traffic flows and their corresponding usable routing paths to detect underutilized links. A link is considered to be underutilized if it is being utilized below a specific threshold relative to its total assigned capacity. In our case we set the upper bound of this threshold to 10% and in the topology marking phase all networks links whose utilization are below this specified threshold are marked as underutilized. The underutilized links are marked by assigning the red color. If any underutilized link is identified, its corresponding traffic flow and routing path is processed by LINK-OPTIMIZATION algorithm. It finds an alternative underutilized-link free routing path from the first detected underutilized link that fulfills its desired QoS and capacity requirements. If the desired path will be found that would be selected and a complete end-to-end new routing path will be constructed. After constructing new path the corresponding traffic flow will be redirected over this new routing path. If no desired alternative routing path will be found the previous path will be remain intact. Finally, the LINK-OPTIMIZATION algorithm computes the set of free links by re-computing and modifying the utilization of old and new paths. A link is considered to be free/extra if its total utilization becomes zero. In the end, the TOPOLOGY-OPTIMIZATION algorithm reconstructs the final and optimized connected network topology by removing the extra link and nodes from the original network topology.

A node is considered to be extra if the sum of its all incident links becomes zero and such a node is also called isolated node.


**Algorithm 1:** Link and Node Removal Considering Residual Capacity and Traffic Demands (LNR-RCTD)
**Inputs:** An undirected connected network *G*(*V*,*E*), where *V* is the set of nodes, *E* is the set of edges, *F* is the set of flows, and *P_fi,j_* is the set of usable paths for each flow *P_fi,j_*.**Begin**1:**for** each 
$f_{i,k}^{j}$ ∈ *F*
**do**2: **for** each *e_l,m_* ∈ 
$UP_{f_{i,k}^{j}}$
**do**3:  **if** color (*e_l_*_,_*_m_*) ⩵ *Red*
**then**4:   *LINK-OPTIMIZATION* (*e_l,m_*, *v_e_l,m__*, 
$f_{i,k}^{j}$, 
$QoSP_{f_{i,k}^{j}}$)5:  **end if**6: **end for**7:**end for**8:*TOPOLOGY- OPTIMIZATION (F_extra_)*


The LNR-RCTD algorithm (see Algorithm 1) is used to find the underutilized links in WMNs enabled with real-time and multimedia services and applications:

1. *Algorithm:* LNR-RCTD algorithm processes for all traffic flows given by set of flows *F*. It then finds underutilized link from the complete end-to-end path specified by $UP_{f_{i,k}^{j}}$ of traffic flow $f_{i,k}^{j}$ ∈ *F*. If a underutilized link is detected it will be processed by *LINK-OPTIMIZATION* algorithm. Finally, *TOPOLOGY-OPTIMIZATION* algorithm constructs the final and optimized connected network topology.
  LINK-OPTIMIZATION algorithm (see Algorithm 2) is used to finds alternative underutilized-link free end-to-end paths that fulfill the desired QoS requirement of flow $f_{i,k}^{j}$. If the desired path from detected link will be found, the algorithm constructs a new or partial new path and assign it to its respective flow $f_{i,k}^{j}$. Finally, algorithm recomputes and modify the utilization of both old and new paths and append links to the set of free links *F_extra_* if and on if their utilization becomes zero.
2. *Algorithm:* The algorithm starts by assigning underutilized link's corresponding node *v_el,m_* to a temporary variable *v*_1_ and then it finds an alternative underutilized-link free path through its all incident links. The loop on line 2 processes till it's finds an alternative path through its all available incident links. Set of all possible paths ${\text{p}}_{{\text{f}}_{\text{i},\text{k}}^{\text{j}}}$ between nodes v_1_ and v_k_ started from link e_l,n_ are computed by PathFinder ( ${\text{f}}_{\text{i},\text{k}}^{\text{j}}$, e_l,n_, v_1_) at line 3. From line 4 to line 11, it operates over a set of paths ${\text{p}}_{{\text{f}}_{\text{i},\text{k}}^{\text{j}}}$ to finds an alternative path. If a required alternative path found, algorithm computes new end-to-end path and redirect its respective flow over that new routing path. Along with this it re-computes capacities utilization of old and new path and construct set of extra link *F_extra_*. At line 12 the subroutine Underutilize (P_l,K_) examines whether the path P_l,K_ contains any further underutilized links or not. From line 13 to line 19, algorithm computes and examines end-to-end delay, PLR and residual capacity of path. If it qualifies all these constraints it will be selected. After the desired path/sub-path selection, algorithm reconstructs its complete end-to-end routing path at line 6 according to Equation (22) and assigns it to its respective flow ${\text{f}}_{\text{i},\text{k}}^{\text{j}}$. Finally, algorithm assign link *e_l,m_* to set of extra links *F_extra_* and re-computes the utilization of old and new paths using *CapRecomp* ( $f_{i,k}^{j}$, *P_l,K_*, $NUP_{f_{i,k}^{j}}$)as follows:
  (20) $$cc_{e_{st}}=cc_{e_{s,t}}+f_{ij}\text{and}cc_{e_{stt}}=cc_{e_{s,tt}}-f_{ij}$$
  where *CC_est_* and *CC_es,tt_* re the capacities of each link belonging to path *P_l,K_* and $NUP_{f_{i,k}^{j}}$, respectively.

**Algorithm 2:** LINK-OPTIMIZATION
**Inputs:** A selected link *e_lm_*, its node *v_elm_*, corresponding flow 
$f_{i,k}^{j}$, and set of Qos parameters 
$QoSP_{f_{i,k}^{j}}$.**Output:** A set of extra links *F_extra_*.**Begin**1:*v_l_* ← *v_el,m_*2:**while**
*e_l,n_* ∈ *I*_(*v_l_*)_ ≠ ∅ & *p_alt_* ≠ 1 **do**3: 
${\text{p}}_{{\text{f}}_{\text{i},\text{k}}^{\text{j}}}\leftarrow \text{PathFinder}(f_{i,k}^{j},e_{l,n},v_{l})$4: **for** each 
$P_{l,k}\in p_{f_{i,k}^{j}}$
**do**5:  **if**
*p_alt_* ⩵ 1 **then**6:   
$NUP_{f_{i,k}^{j}}=PP_{il-1,j}\cup P_{l,k}$7:   Assign (
$f_{i,k}^{j}$, *P_l,K_*)8:   *CapRecomp* (
$f_{i,k}^{j}$, *P_l,K_*, 
$UP_{f_{i,k}^{j}}$)9:   *F_extra_* ← *e_l,m_*10:   break11:  **end if**12:  **else if** (*Underutilize (P_l,K_) ⩵0*) **then**13:   Compute *Delay of P_l,K_ by using*
Equation (4)14:   **if** (
$\tau {\left(t\right)}_{P_{L,K}}\leq d_{f_{i,k}^{j}}$) **then**15:    Compute Loss of *P_l,K_* by using Equation (5)16:    **if** (
$l{\left(t\right)}_{P_{L,k}}\leq PLR_{f_{i,k}^{j}}$) **then**17:     **for** each *e_l_* ∈ *P_l,K_*
**do**18:      Compute Residual-Cap by using Equation (1)19:      **if** (
$RC_{e_{l}}\leq f_{i,k}^{j}$
**then**20:      break21:      **end if**22:     **end for**23:      *p_alt_* ← 124:     **end if**25:    **end if**26:  **end if**27: **end for**28:**end while**
  TOPOLOGY-OPTIMIZATION removes the set of extra links *F_extra_* ⊆ *E* from the origional network topology and reconstruct the optimized end-to-end connected topology. Final optimized topology *G′*(*V′*,*E′*) ⊆ *G*(*V*, *E*) also ensures the sufficient redundency to keep the origional design goal of WMNs.
3. *Algorithm:* From line 1 to 3 the algorithm removes the set of extra links *F_extra_* ⊆ *E* from the original topology. From line 4 to line 6 it examines the isolation of all WMRs and if some WMRs are found to be isolated, it will be added to the set of extra WMRs *V_extra_*. Finally, from line 8 to line 11 it removes all isolated WMRs. Note: A WMR *v_j_* is said to be isolated if the sum of its all incident links will be zero.

### 5.2. LNR-RCTD Algorithm Illustration

**Figure 2.**
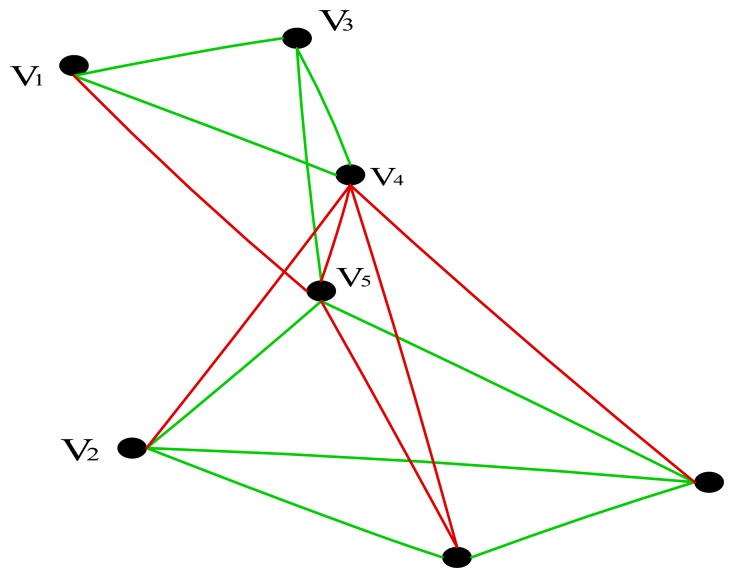
A small connected network topology.

We applied our algorithm on a simple network topology, as shown in Figure 2, to demonstrate it step by step working. We explain the LNR-RCTD in action by taking the example of single flow say $f_{6,1}^{1}$ and let's assume its associated usable path is $UP_{f_{6,1}^{1}}=\left\{v_{6},v_{7},v_{4},v_{3},v_{1}\right\}$. This routing path is used to transmit data traffic from source node *v*_6_ to destination node *v*_1_.The loop at line 1 of algorithm 1 operates for only this selected single flow. The loop at line 2 processes all the links in the path $UP_{f_{6,1}^{1}}$ and each link will be tested to determine whether it is underutilized or not. This test is carried out by a subroutine at line 3. In our mentioned example, link *e*_7,4_ is detected as underutilized (This fact is evident from Figure 2, in which all the underutilized links are shown in red). After detecting underutilized link *e*_7,4_ on node *v*_7_ the LINK-OPTIMIZATION algorithm is being activated at line 4. Before going into further discussion, we clarify an important concept here. $UP_{f_{6,1}^{1}}=\left\{{\text{v}}_{\text{i}},{\text{v}}_{\text{i}+1},\ldots ,{\text{v}}_{\text{n}},{\text{v}}_{\text{m}}\ldots {\text{v}}_{\text{k}}\right\}=\left\{{\text{v}}_{6},{\text{v}}_{7},{\text{v}}_{4},{\text{v}}_{3},{\text{v}}_{1}\right\}$ is the usable path for flow $f_{6,1}^{1}$. This path is further divided into two parts, $UNUP_{f_{6,1}^{1}}=\left\{{\text{v}}_{\text{n}},{\text{v}}_{\text{m}}\ldots {\text{v}}_{\text{k}}\right\}=\left\{{\text{v}}_{7},{\text{v}}_{4},{\text{v}}_{3},{\text{v}}_{1}\right\}$ that is the unused portion of the path starting from the red (underutilized) link, and *PP_i,l_*_−1_ = {v_i_,v_i+1_,…, v_n−1_} = {v_6_} is the previous portion of the usable path before the occurrence of the red link. This concept will be used in constructing the new usable underutilized link-free path for flow $f_{6,1}^{1}$.

In the *LINK-OPTIMIZATION* algorithm a loop at line 2 processes all the incident links of node v_7_ until it finds an alternate path for $UNUP_{f_{6,1}^{1}}$. In the mentioned example, the incident links to node v_7_ are e_7,2_ and e_7,5_.The subroutine *PathFinder* at line 3 will computes all available paths starting from node *v*_7_ and ending at destination node *v*_1_for flow $f_{6,1}^{1}$. We only compute those paths that are loop-free, meaning they do not contain the original source node v_6_ of flow $f_{6,1}^{1}$. As in the mentioned example there are only two incident links at node v_7_, Thus, the available paths are collectively represented by $p_{f_{6,1}^{1}}$ as shown in Table 3.

The loop at line 4 processes all paths shown in Table 3 until it finds required alternative path. At line 12, subroutine Underutilize tests whether the current path contains any further underutilized links or not. If a path contains any underutilized link, it will not be selected. Table 4 shows underutilized link-free paths. Now onward, these are the only paths of interest.

From line 13 to line 19 the algorithm ensures whether the current path fulfills the QoS requirements (*i.e.*, bandwidth, delay and PLR) of flow $f_{6,1}^{1}$. Table 5 represents the available paths and their respective QoS parameters.

The value of residual capacity is as follows:

(21) $$\text{Residual}-\text{Cap}=\mathrm{Min}(e_{i,j},e_{j,k},\ldots ,e_{l,n})$$

where 384 kbps of data rate, 400 ms of end-to-end delay and less than 2% of PLR are the QoS parameters for flow $f_{6,1}^{1}$. Under such QoS constraints, the first path is not selected while the second path fulfills the desired QoS constraints therefore, that path will be selected. Thus, the new usable end-to-end path $NUP_{f_{6,1}^{1}}$ is reconstructed according to Equation (22):

(22) $$NUP_{f_{i,k}^{j}}=PP_{il-1,j}\cup P_{l,K}$$

$NUP_{f_{6,1}^{1}}=\left\{v_{6}\to v_{7}\to v_{2}\to v_{5}\to v_{3}\to v_{4}\to v_{1}\right\}$. After constructing the new path, the flow $f_{6,1}^{1}$ is redirected on this new path and residual capacities over both the newly selected path $NUP_{f_{6,1}^{1}}$ and the unused portion of the old path $UNUP_{f_{6,1}^{1}}$ are recomputed. The underutilized link *e*_7,4_ is added to the set of extra links *F_extra_* if its used capacity becomes zero. Finally, the *TOPOLOGY-OPTIMIZATION* algorithm is being activated. It simply removes all the links in the set of extra links *F_extra_* and then tests the isolation of all network nodes. If a node is found to be isolated, it will be added to the set of extra nodes *V_extra_* and finally *TOPOLOGY-OPTIMIZATION* also removes all nodes in the set *V_extra_*.The final result of *TOPOLOGY-OPTIMIZATION* is an optimized connected network topology *G′*(*V′*,*E′*) ⊆ *G*(*V*, *E*).

## 6. Complexity Analysis

In this section, we present the time complexity of the proposed heuristic LNR-RCTD algorithm. The overall complexity analysis is divided into three parts to maintain uniformity with our proposed heuristic. First, we analyze the time complexity of line 1 and 2 of the algorithm 1 as shown below. The time complexity of line 1 and 2 is $\sum_{i=1}^{\alpha n}1=\alpha n$ and $\sum_{j=1}^{N}1=m$, Respectively. Where *αn* represent the total number of network flows and *N* is the average path length. Thus, the complexity of this portion is as follows:

(23) $$\sum_{i=1}^{\alpha n}\sum_{j=1}^{N}c_{0}=c_{0}.\alpha n.N$$

Second, we analyze the time complexity of the *LINK-OPTIMIZATION* algorithm. Its time complexity is mainly based on line 2, 3, and 4 while the remaining portion of Algorithm 2 is comprised of a few subroutines that have almost constant time and do not contribute much to the overall complexity. Thus, the time complexity of Algorithm 2 is as follows:

(24) $$\sum_{k=1}^{z}NM_{\text{paths}}\sum_{l=1}^{M_{\text{paths}}}C=z.NM_{\text{paths}}.CM_{\text{paths}}=CNz{\left(M_{\text{paths}}\right)}^{2}$$

Where *z* is the average degree of the node and *NM_paths_* is the average path length multiplied by the total number of paths. Hence, *NM_paths_* is the time complexity of path finding and is calculated in APAC [52]. The loop at line 4 runs approximately *M_paths_* times and *C* is a constant representing the time complexity of the portions comprised of subroutines. Finally, we analyzed the time complexity of the *TOPOLOGY-OPTIMIZATION* algorithm. Its time complexity is mainly based on line1, 4, and 9. Thus, the time complexity of Algorithm 3 is as follows:

(25) $$\sum_{e_{i}}^{E_{\text{extra}}}c_{1}+\sum_{v}^{V}c_{2}+\sum_{v_{i}}^{V_{\text{extra}}}c_{3}=c_{1}E_{\text{extra}}+c_{2}v+c_{3}V_{\text{extra}}$$

Equation (26) is the result of combing Equations (23)–(25):

(26) $$TC=\left(c_{0}.\alpha n.N\right).CNz{\left(M_{\text{paths}}\right)}^{2}+\left(E_{\text{extra}}+v+V_{\text{extra}}\right)$$

where *TC* is the time complexity of LNR-RCTD. Since, we are calculating the Big-Oh time complexity. Therefore, we eliminate the least significant factors and only select the most significant factors those mainly contribute in the time complexity of LNR-RCTD. Thus, finally we get:

(27) $$TC=O\left(C.z\alpha n.N^{2}{M_{\text{paths}}}^{2}\right)$$

Equation (27) shows that the time complexity of the proposed heuristic algorithm is polynomial time.


**Algorithm 3:** TOPOLOGY-OPTIMIZATION
**Inputs:** A set of extra links *F_extra_* and set of vertices V**Output:** A connected sub-graph *G*′(*V*′, *E*′).**Begin**1:**for** each *e_extra_* ∈ *F_extra_*
**do**2: *Remove* (*e_extra_*)3:**end for**4:**for** each *v_j_* ∈ *V*
**do**5: **if** (Isolated (*v_j_*) = = True) **then**6:  *V_extra_* ← *v_j_*7: **end if**8:**end for**9:**for** each *v_extra_* ∈ *V_extra_*
**do**10: *Remove* (*v_extra_*)11:**end for**


## 7. Simulation Study

### 7.1. Methodology and Simulation Settings

We implement both the ILP model and LNR-RCTD in MATLAB and evaluate their performance with simulations. In our simulation setup, clients (*i.e.*, source, destination) nodes are fixed, total client source nodes are set to be 100, and each client source node generates multimedia traffic that has to be transmit to its corresponding destination node. Table 6 represents the QoS requirements of multimedia traffic flows used in our simulation. Table 6 is derived from 3GPP TS 22.105 [54]. A random backbone mesh topology with different node density is generated within a 1000 × 1000 area of two dimensional regions. The transmission range of each node is set to be 300 m, and if the Euclidean distance between two mesh backbone nodes or WMRs is less than the transmission range, an edge is being generated and channel is assigned based on channel interference model. We used IEEE802.11 a/b hybrid radios to utilize orthogonal channel of both IEEE 802.11 b and a. Therefore, number of radios available to each mesh node is directly proportional to the number of available orthogonal channels to that node. Capacity of each link is set to be 100 Mbps, and each link is marked as underutilized if its aggregated utilization is below 10% of the total assigned capacity. Our proposed solutions only operate on a connected topology. Therefore, before applying our solutions the overall connectivity of the mesh topology is assured. QoS-provisioned paths for each pair of source and destination nodes are computed using APAC.

### 7.2. Simulation Results

The following figures demonstrate the working and performance of LNR-RCTD algorithm.

**Figure 3.**
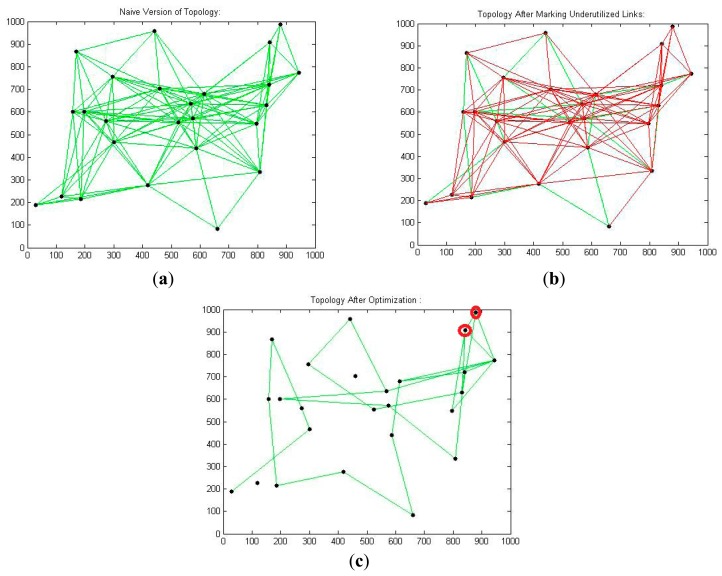
(**a**) 25 nodes' naive topology; (**b**) 25 nodes' marked topology; (**c**) 25 nodes' optimized topology.

Figure 3a–c represent the naive, underutilize-marked, and optimized versions of the mesh topology, respectively. In Figure 3c, the red circled nodes are those nodes whose all incident links are marked as underutilized, as shown in Figure 3b, but after applying LNR-RCTD, optimization these nodes could not be released. This is due to our simulation's computational limitations, as we only compute and store the first 100 alternative paths for each marked link from its parent node (where underutilized link occur) and then compute the QoS parameters of each path one by one and compare them with the actual QoS demands. If some alternative path is found, then that flow will be shifted on new path, otherwise, that marked link remains intact.

**Figure 4.**
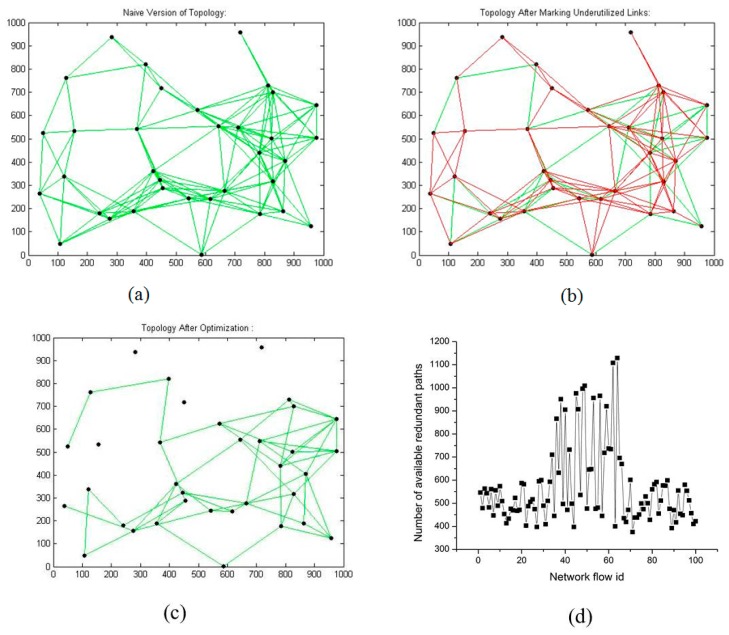
(**a**) 35 nodes' naive topology; (**b**) 35 nodes' marked topology; (**c**) 35 nodes' optimized topology; (**d**) Path redundancy.

**Figure 5.**
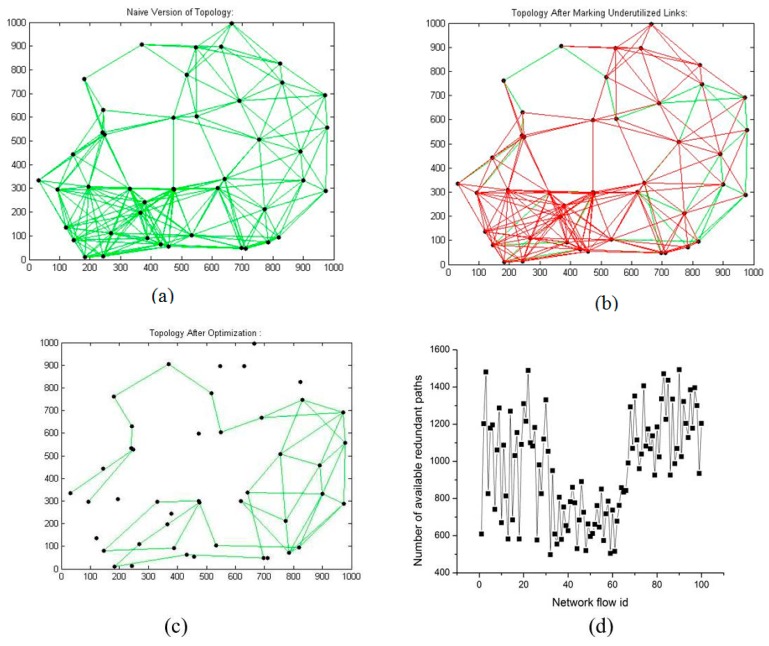
(**a**) 45 nodes' naive topology; (**b**) 45 nodes' marked topology; (**c**) 45 nodes' optimized topology; (**d**) Path redundancy.

Figure 4a–d represent the naive, underutilized-marked, optimized, and redundant paths after optimization, respectively. In Figure 4b, the bold and red links shows the underutilized links. After applying LNR-RCTD optimization, four nodes and 87 links are identified. In Figure 4c, the nodes' having no incident links are identified as extra nodes'. Redundancy is one of the major design objectives in WMNs, and our LNR-RCTD optimization also preserves the redundancy of WMNs by only removing the extra resources. Figure 4d represents the redundant available paths for each network flow ID after optimization. Figure 5a–d represent the naive, underutilized-marked, optimized, and redundant paths after optimization, respectively. After applying LNR-RCTD optimization, 11 extra nodes and 171 links are identified as extra.

**Figure 6.**
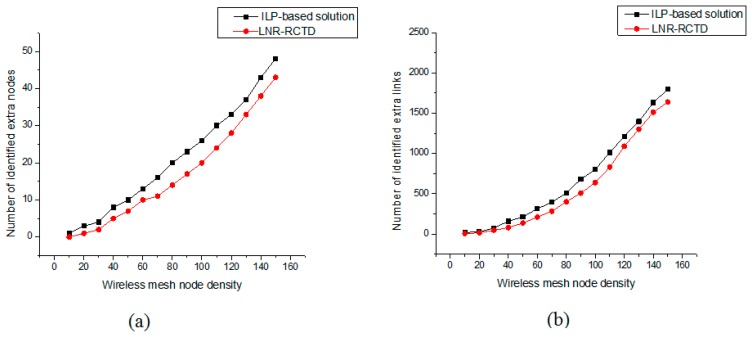
(**a**) Identification of extra mesh nodes; (**b**) Identification of extra wireless links.

The following Figure 6a,b represent the identified extra wireless mesh backbone nodes and links, respectively. The presented results are the averages of 100 simulations for the same node density. The Figure 6a,b demonstrate that a lot of physical resources are being wasted. From these figures we can calculate % of wasted resources for 100 nodes. The calculation is given as follows:

(28) $$\begin{array}{c} \text{Numbers of extra nodes}=\frac{20}{100}\times 100=20\%\\ \text{Numbers of extra links}=\frac{650}{2700}\times 100=24.07\% \end{array}$$

where 20 and 650 is the numbers of identified extra nodes and links, respectively. 2700 is the total numbers of links before topology optimization. From the above calculations it is clear that more than 20% of physical layer resources are being wasted by QoS provisioning routing and random topology deployment.

Moreover, the above figures show that ILP performs better than LNR-RCTD. This is due to two reasons: (1) ILP always gives the optimal results or the upper bound of the solution; (2) in LNR-RCTD, we only compute and store the first 100 available alternative paths against each marked link. We only exploit these paths to compute the QoS parameters of each path one by one and compare them with the actual QoS demands. If some alternative path is found, the flow will be rerouted on newly computed path otherwise marked link remains intact while ILP explores all available solutions.

### 7.3. Multipath vs. Single Routing

In the single path routing, the multimedia traffic flow between source and destination pairs should be routed on the single path. Thus, in single path routing problem the task is to find a feasible path between source and destination pairs that fulfills the QoS requirements of multimedia traffic flow as well as fulfills the capacity constraint. Single path routing approach may not fully utilize the assigned capacities.

In multipath routing, the multimedia traffic flow is divided into multiple sub-flows. In multipath routing problem the task is to find the set of feasible paths that fulfills the QoS requirements of each multimedia sub-flow along with their capacity constraints. Multipath routing scheme may provide more effective bandwidths utilization.

## 8. Conclusions

Wireless mesh networking is a promising technology that has tremendous capabilities to support multimedia and QoS-demanding applications and services. To enable multimedia and QoS-demanding applications and services over WMNs, QoS provisioning routing is required, which is an emerging field. Available QoS provisioning routing protocols uses greedy parameters to find and select end-to-end QoS-provisioned paths. Furthermore, the deployment of WMNs is random. Thus, QoS provisioning routing and random topology deployment causes severe wastage of physical layers resources, ultimately increasing the deployment and operating costs.

In this paper, we have proposed a novel ILP-based optimization solution and a polynomial time heuristic algorithm to reconstruct an optimized mesh backbone topology with a minimum number of wireless communication links and backbone WMRs. Our simulation study shows that more than 20% of physical layer resources are being wasted by QoS provisioning routing and random topology deployment. Our proposed heuristic LNR-RCTD algorithm achieved near-optimal results and the time complexity of LNR-RCTD is polynomial. Our reconstructed optimized backbone mesh topology also maintains a specific level of redundency, which is the core design objective of WMNs. In this paper, we only considered single-path routing between source and destination pairs. This work can be further extended to multipath routing between source and destination pairs.

## Figures and Tables

**Table 1.** Notations.

*v_i_*	Wireless mesh node *i*
*v_n_*	Total numbers of network nodes
*R_vi_*	Numbers of IEEE802.11 hybrid radio available to *v_i_*
*c_k_*	Total number of orthogonal channels
*e_i,j_*	Link from node *v_i_* to *v_j_*
*e_m_*	Total numbers of network edges
*I_R_*	Interference range of communication node *v_i_*
*d_vi,vk_*	Euclidean distance between nodes *v_i_* and *v_k_*
*k_max_*	Maximum number of orthogonal channels
*f_y_*	Total number of network flows
*cc_em_*	Channel capacity assigned to link *e_m_*
*r_em_*	Residual capacity of link *e_m_*
*p_z_*	Total number of alternative paths for flow *f_y_*
*d_fy_*	Affordable delay limit of flow *f_y_*
*pl_fy_*	Affordable PLR limit of flow f_y_
*RC_ei,j_*	The residual capacity of link *e_i,j_*
*CC_ei,j_*	Total assigned capacity to link *e_i,j_*
*f_o_*	The amount of capacity used by traffic flow *f_o_*.
${F^{\prime }}_{e_{i,j}}$	The set of flows traversing from link *e_i,j_*

**Table 2.** Notations for LNR-RCTD algorithm.

$f_{i,k}^{j}$	*j^th^* flow from node *v_i_* to *v_k_*
$p_{f_{i,k}^{j}}$	The set of all available paths from node *v_i_* to node *v_k_* for flow $f_{i,k}^{j}$
$UP_{f_{i,k}^{j}}$	The used path by flow $f_{i,j}^{k}$ starts from node *v_i_* and ends at *v_k_*
$UNUP_{f_{i,k}^{j}}$	Unused portion of the path for flow $f_{i,k}^{j}$
$NUP_{f_{i,k}^{j}}$	New usable path for flow $f_{i,k}^{j}$
$PLR_{f_{i,k}^{j}}$	The maximum affordable PLR for flow $f_{i,k}^{j}$
$d_{f_{i,k}^{j}}$	The maximum affordable delay limit for flow $f_{i,k}^{j}$
$QoSP_{f_{i,k}^{j}}$	Vector contains QoS parameters of $f_{i,k}^{j}$
*PP_i,l_*_−1_	The portion of usable path before the occurance of an underutilized link.
[v_1_, v_2_, v_3_, v_4_]	A path formed by the sequence of nodes v_1_, v_2_, v_3_, v_4_.

**Table 3.** $p_{f_{7,1}^{1}}$ for flow $f_{6,1}^{1}$ from node *v*_7_ to node *v*_1_.

[v_7_, v_2_, v_5_, v_1_], [v_7_, v_2_, v_5_, v_4_, v_1_], [v_7_, v_2_, v_5_, v_4_, v_3_, v_1_],
[v_7_,v_2_, v_5_, v_3_, v_1_], [v_7_, v_2_, v_5_, v_3_, v_4_, v_1_], [v_7_, v_2_, v_4_, v_1_],
[v_7_, v_2_,v_4_, v_5_, v_1_], [v_7_, v_2_, v_4_, v_5_, v_3_, v_1_], [v_7_, v_2_, v_4_, v_3_, v_1_],
[v_7_, v_2_,v_4_, v_3_, v_5_, v_1_], [v_7_, v_5_, v_1_], [v_7_, v_5_, v_4_, v_1_],
[v_7_, v_5_, v_4_, v_3_, v_1_], [v_7_, v_5_, v_3_, v_1_], [v_7_, v_5_, v_3_, v_4_, v_1_],
[v_7_, v_5_, v_2_, v_4_, v_1_], [v_7_, v_5_,v_2_, v_4_, v_3_, v_1_]

**Table 4.** Underutilized link-free paths for $f_{6,1}^{j}$ from *v*_7_.

[v_7_,v_2_, v_5_, v_3_, v_1_], [v_7_, v_2_, v_5_, v_3_, v_4_, v_1_],
[v_7_, v_5_, v_3_, v_1_], [v_7_, v_5_, v_3_, v_4_, v_1_]

**Table 5.** Residual-cap delay and loss associativity.

**Path**	**Residual-Cap (Mbps)**	**Delay (ms)**	**Packet Loss Rate (%)**
[v_7_,v_2_, v_5_, v_3_, v_1_]	0.3	150	0.02
[v_7_, v_2_, v_5_, v_3_, v_4_, v_1_]	8.0	200	0.009
[v_7_, v_5_, v_3_, v_1_]	0.5	100	0.075
[v_7_, v_5_, v_3_, v_4_, v_1_]	5.0	150	0.02

**Table 6.** QoS requirements.

**Type**	**Application**	**Bitrate (kb/s)**	**Delay (s)**	**PLR**
Audio (CBR)	Speech, high quality music	128	<2	<1%
Video (CBR)	Real-time video, surveillance	384	<2	<2%
Data	Bulk data transfer	<384	NA	0%
